# Supplementary material for: Improving HIV testing, linkage, and retention in care among South African men through U=U messaging: A study protocol for two sequential hybrid type 1 effectiveness- implementation randomized controlled trials
Source: Res Sq. 2023 Oct 4:rs.3.rs-3349696. Preprint. [Version 1] doi: 10.21203/rs.3.rs-3349696/v1 (PMC10602079; doi:10.21203/rs.3.rs-3349696/v1)
Supplement: Supplement 1 [file NIHPPRS3349696V1-supplement-1.pdf]

Supplementary Files

This is a list of supplementary files associated with this preprint. Click to download.

- 6.SupplementalFigure1.docx
- 7.SupplementalTables12.docx
